# Dexmedetomidine for preventing postoperative delirium in neurosurgical patients: a meta-analysis of randomized controlled trials

**DOI:** 10.1016/j.bjane.2025.844662

**Published:** 2025-07-10

**Authors:** Virgilio Astori, Bruno Pandolfi Arruda, Pedro Guimarães Marcarini, Lucas Destefani Natali, Marcos Sampaio Meireles, Daniele Fernandes Holanda

**Affiliations:** a Escola Superior de Ciências da Santa Casa de Misericórdia de Vitória, Vitória, ES, Brazil; b Universidade Federal do Amazonas, Manaus, AM, Brazil

**Keywords:** Delirium, Dexmedetomidine, Neuroprotective agents, Neurosurgery

## Introduction

Delirium is an acute, fluctuating disturbance of attention and cognition, often with altered consciousness and perception. Postoperative Delirium (POD) commonly occurs within one week of surgery or before discharge. Its prevalence after neurosurgery ranges from 12% to 26%, averaging 19%.^1^

Although dexmedetomidine, a selective alpha-2 adrenergic receptor agonist, is frequently recommended for POD prevention, especially in neurological surgeries, published evidence remains inconsistent. Recent studies suggest that, while dexmedetomidine may reduce the incidence of delirium in certain cases, its efficacy is not consistently observed across various surgical procedures.

Given these inconsistencies and the substantial impact of POD on patient outcomes, it is crucial to clarify the role of dexmedetomidine in preventing POD. To address this need, we performed a comprehensive meta-analysis of randomized controlled trials focusing specifically on neurosurgical patients. This study aims to evaluate the efficacy of dexmedetomidine in reducing POD incidence, offering clearer guidance for its application in neurocritical care and bridging existing gaps in the current body of knowledge.

## Methods

This systematic review and meta-analysis were conducted and reported in accordance with the Cochrane Handbook for Systematic Reviews of Interventions^2^ and the Preferred Reporting Items for Systematic Reviews and Meta-Analyses (PRISMA) guidelines.^3^ The prospective meta-analysis project was registered on PROSPERO under protocol CRD42024577345. We considered studies eligible for inclusion if (1) They were RCTs; (2) Patients had undergone neurosurgical procedures; (3) They compared dexmedetomidine versus placebo; (4) They presented data regarding any of the clinical outcomes of interest. Exclusion criteria encompassed: (1) Case reports, review articles, and observational studies; (2) Lack of sufficient data for analysis.

A systematic search was conducted across PubMed, Embase, Scopus, ClinicalTrials.gov and the Cochrane Central Register of Controlled Trials from their inception up to March, 2025. The following MeSH (Medical Subject Headings) terms were used for the MEDLINE search and were adapted as needed for other databases. The full search strategy can be found in Supplementary Table 1. The remaining relevant literature was independently screened and evaluated for inclusion in the systematic review by title and/or abstract by two authors (V.A. and B.P.A.). The first reviewer (V.A.) screened the studies for duplicates, while the second reviewer (B.P.A.) assessed the studies against the eligibility criteria. The full texts of potentially eligible studies were then retrieved and reviewed for further selection by both authors. Any disagreements were resolved through consensus between the two reviewers.

The primary outcome was the incidence of POD. We extracted the incidence rates based on the assessments and criteria utilized in each individual study. Two authors (B.P.A. and V.A.) independently extracted data according to predetermined search criteria and performed quality assessments. The risk of bias in randomized studies was evaluated using version 2 of the Cochrane Risk of Bias assessment tool.^4^ The Risk Ratio (RR) was utilized to compare the intervention effect for dichotomous endpoints, presented with 95% Confidence Intervals (CIs). To assess heterogeneity, we employed the Cochrane *Q* test and I² statistics, considering p < 0.10 and I² > 40% as significant indicators of heterogeneity. We also performed sensitivity analyses with restriction of the study with the highest weight. The outcome was analyzed using a random-effects model. The statistical analysis was performed using RevMan version 8.1.1.^5^

## Results

Our systematic search yielded 71 potential articles. After removing duplicates and studies that did not meet the eligibility criteria, nine studies were retrieved and reviewed in full for possible inclusion. Of these, three met all inclusion criteria and were included in our analysis, comprising a total of 526 patients (Supplementary Fig. 1). Among them, 264 patients (50.2%) received dexmedetomidine. The main characteristics of the included studies are detailed in Table 1.

**Table 1:** Characteristics of studies included in meta-analysis.

Study	Population	No, of Patients DEX/Placebo	Female, % DEX/Placebo	ASA class (I/II/III/IV) DEX ‒ Placebo	Delirium assessment tool^a^	DEX dosage	Surgery type
Chen 2021	Patients > 20 y who had undergone elective cranial surgery for brain tumor resection, aneurysm clipping, intracranial bypass procedure, and microvascular decompression, between April, 2017 ‒ April, 2020	80/80	62.5/58.7	5/48/27/0 ‒ 3/53/24/0	ICSDC	0.5 μg.kg^-1^.h^-1^ before start of surgery and maintained until end of surgery	Brain tumor resection, aneurysm clipping, intracranial bypass procedure, and microvascular decompression
Li 2022	Patients with frontotemporal brain tumors > 18 yr old who were scheduled for elective craniotomy with general anesthesia. MMSE > 20	130/130	44.6/50.8	6/77/45/2 ‒ 6/66/57/1	CAM-ICU	Loading dose: 0.6 μg.kg^-1^.h^-1^; maintenance: 0.4 μg.kg^-1^.h^-1^ until dural close	Brain tumor resection
Tang 2018	Patients with ASA I to IV, 18‒70 y, Glasgow coma scale > 11, Hunt-Hess I‒III and embolization of intracranial aneurysms	54/52	44/50	16/34/2/2 ‒ 18/32/1/1	Modified CAM-S	1 μg.kg^-1^ for 15 minutes; maintenance: 0.3 μg.kg^-1^.h^-1^ until end of surgery	Intracranial aneurysm embolization

Y, Years; ASA, American Society of Anesthesiologists; MMSE, Mini-Mental State Examination; RCT, Randomized Controlled Trial; DEX, Dexmedetomidine; ICSDC, Intensive Care Delirium Screening Checklist; CAM-ICU, Confusion Assessment Method for Intensive Care Unit; CAM-S, Confusion Assessment Method.

a Description of each delirium assessment tool in Supplementary Table 2.

**Figure 1:**
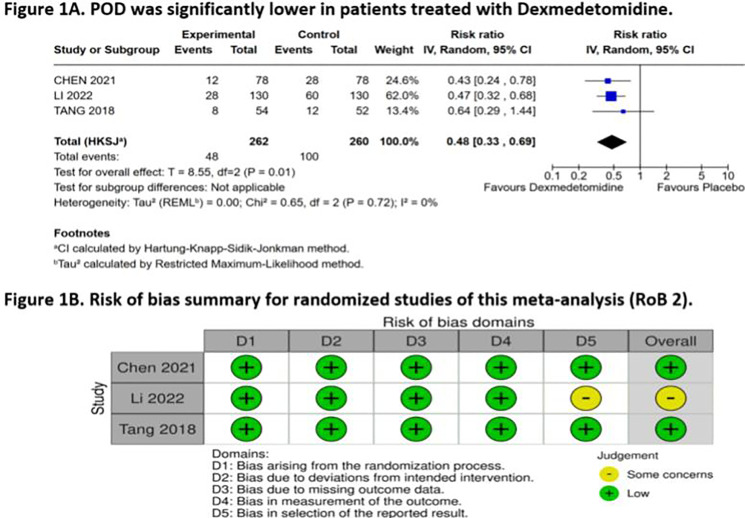
(A) Forest plot of studies examining outcomes between patients in dexmedetomidine intervention and saline placebo; (B) Risk of bias of this meta-analysis.

The analysis of the incidence rates of POD (RR = 0.48; 95% CI 0.35‒0.64; p < 0.00001; I² = 0%; Fig. 1A) revealed a statistically significant reduction in the dexmedetomidine group compared to the placebo group. In the sensitivity analysis, the exclusion of the study with the highest weight (59.9%) did not significantly affect the overall result (RR = 0.49; 95% CI 0.31‒0.80; p = 0.004; I² = 0%; Supplementary Fig. 2). Moreover, the results illustrate a potential 52% reduction in POD risk, with an Absolute Risk Reduction (ARR) of 20.1% (95% CI 13.9%‒25.1%), leading to a Number Needed to Treat (NNT) of 5 (95% CI 4‒8) patients.

Among the three studies evaluated, one study^6^ was found to have some concerns, specifically in the domain of bias in the selection of the reported results (Fig. 1B). The other two studies^7^^,^^8^ were identified as having an overall low risk of bias across all domains. Notably, no studies were found to be at high risk of bias.

## Discussion

This systematic review and meta-analysis, encompassing three studies with a total of 526 patients, is the first to assess the efficacy of dexmedetomidine in reducing POD in neurosurgical patients. The primary finding demonstrates a significant reduction in POD rates among patients treated with dexmedetomidine.

Dexmedetomidine exerts neuroprotective effects mediated by brain-derived neurotrophic factor, which inhibits Nucleotide-binding domain-Like Receptor Protein-3 (NLRP3) inflammasome, reduces macrophage infiltration, microglial migration, and neurological damage. It also modulates autophagy and decreases microtubule-associated Light Chain-3 (LC3), Beclin-1, and Nuclear Factor kappa-B (NF-κB).^9^ These pathways lower pro-inflammatory cytokines, contributing to neuroinflammation control.^10^

All studies included in the meta-analysis^6^, ^7^, ^8^ consistently demonstrated a lower incidence of POD in the patients who received dexmedetomidine compared to those in the placebo group. Two studies^7^^,^^8^ also assessed the severity of POD in addition to its incidence. The results revealed that the dexmedetomidine group not only exhibited fewer cases but also experienced milder forms of delirium compared to the control group. These findings suggest that dexmedetomidine may offer a dual benefit in mitigating the intensity of POD.

Our findings demonstrate a statistically significant reduction in the risk of POD among patients receiving dexmedetomidine compared to those given placebo. The pooled risk ratio (RR = 0.48; 95% CI 0.35‒0.64; p < 0.00001; I² = 0%; Fig. 1A) indicates a 52% reduction in the incidence of delirium. This finding is further supported by the absence of significant heterogeneity among the included studies, suggesting consistency in the beneficial effects of dexmedetomidine across different patient populations and study designs.

The dosage of dexmedetomidine varies and is presented in different ways in the literature. It can be administered as a bolus (i.e., a single dose), as an infusion or as a combination of bolus followed by infusion, which is commonly continued during the postoperative period. A variable dose regimen based on the duration of surgery is also described, allowing adjustments to the dosage regimen in accordance with the anesthesiologist or patient-specific factors such as age or weight. Doses are commonly categorized as low (0‒0.49 μg.kg^-1^), medium (0.5‒0.99 μg.kg^-1^) and high (≥ 1 μg.kg^-1^).

Regarding safety, across all included studies,^6^, ^7^, ^8^ the main adverse events associated with dexmedetomidine administration were clinically significant bradycardia and hypotension. Nevertheless, the use of dexmedetomidine was associated with reduced mortality and shorter hospital length of stay, suggesting that these hemodynamic effects likely had minimal or uncertain clinical impact. These findings highlight the need for careful perioperative management, with particular attention to potential effects on hemodynamic stability.

Our meta-analysis has several notable limitations that should be carefully considered. First, the small number of included studies significantly limits the generalizability and robustness of the findings. Although statistical analysis demonstrated consistency in the results, this cannot fully compensate for the limited dataset and its inherent weaknesses. The small sample size increases the risk of overestimating the effect size and reduces the reliability of the conclusions.

Furthermore, varying tools were used across the studies to assess POD (Supplementary Table 2), leading to potential measurement inconsistencies. To manage this variability, we categorized patients dichotomously as either experiencing delirium or not, without accounting for differences in severity or duration.

Additionally, differences in neurosurgical indications, surgical techniques, and perioperative care practices across studies introduce further heterogeneity that may have influenced the outcomes.

These limitations highlight the need for caution when interpreting the results and underscore the importance of future larger-scale studies with standardized assessment methods to confirm the role of dexmedetomidine in reducing POD in neurosurgical patients.

This meta-analysis of randomized clinical trials shows that dexmedetomidine is associated with a significant reduction in the incidence of POD in neurosurgical patients. Although these results support its potential role in improving perioperative outcomes, the underlying mechanisms remain unclear and the adverse effects uncertain, making it a likely obstacle to implementation in clinical practice.

Despite its promising potential, there is a clear need for larger multicenter trials employing standardized delirium assessments and comprehensive safety evaluations regarding its applicability. Future research should aim to determine whether the observed reduction in delirium stems from specific neuroprotective mechanisms or from ancillary factors, such as optimized sedation strategies and modulation of inflammatory responses. Additionally, studies should comprehensively assess dexmedetomidine across diverse clinical settings.

## Research data availability

Data sharing does not apply to this article as no new data were created or analyzed in this study.

## Authors’ contributions

Virgilio Astori: Responsible for conceptualization; data curation; formal analysis; investigation; methodology; project administration; writing-original draft; writing-review & editing.

Bruno Pandolfi Arruda: Responsible for conceptualization; formal analysis; investigation; methodology; writing-original draft.

Pedro Guimarães Marcarini: Responsible for conceptualization; formal analysis; investigation; writing-original draft; writing-review & editing.

Lucas Destefani Natali: Responsible for conceptualization; formal analysis; investigation; writing-original draft; writing-review & editing.

Marcos Sampaio Meireles: Responsible for supervision.

Daniele Fernandes Holanda: Responsible for supervision and writing-review & editing.

All authors have read and approved the final version submitted and take public responsibility for all aspects of the work.

## Fundings

No funding was received for this work.

## Conflicts of interest

The authors declare that they have no competing financial interests or personal relationships that could have influenced the work reported in this paper.

## Appendix Supplementary materials

Image, application 1

## Footnotes

Supplementary material associated with this article can be found in the online version at doi:10.1016/j.bjane.2025.844662.
