# Supplementary material for: Dexmedetomidine for preventing postoperative delirium in neurosurgical patients: a meta-analysis of randomized controlled trials
Source: Braz J Anesthesiol. 2025 Jul 10;75(5):844662. doi: 10.1016/j.bjane.2025.844662 (PMC12539297; doi:10.1016/j.bjane.2025.844662)

**BJAN-D-24-00675_Supplementary Material**

**Supplementary Table 1** Full search strategy used in this meta-analysis.

| **Search strategy** | (DEX OR dexmedetomidine) AND (placebo OR “saline placebo” OR saline OR “saline infusion” OR “saline 0.9%”) AND (delirium OR confusion OR “Emergency Services, Psychiatric” [Mesh]) AND (neurosurgery OR “head surgery” OR “spinal surgery”) |
| --- | --- |

**Supplementary Table 2** Characteristics and criteria of delirium assessment tools used in the included studies.

| **Instruments** | **Description** |
| --- | --- |
| **ISCDC (Intensive Care Delirium Screening Checklist)** | Eight clinical features are assessed: altered level of consciousness, inattention, disorientation, hallucinations/delusions/psychosis, psychomotor agitation or retardation, inappropriate speech or mood, sleep/wake cycle disturbance, and symptom fluctuation, with one item for each feature. Each item is rated as either “obvious manifestation” (1) or “no manifestation or no assessment possible” (0). The worst score in each domain is counted toward the total score, which is calculated at the end of each shift. The sum of all domain scores represents the total score. A score of ≥4 indicates delirium, while a score of 1–3 indicates subsyndromal delirium. |
| **CAM – ICU (Confusion Assessment Method for Intensive Care Unit)** | The diagnosis of delirium is based on the presence of two major criteria (acute or fluctuating onset and inattention) and at least one minor criterion (disorganized thinking or altered level of consciousness). |
| **MODIFIED CAM-S (Confusion Assessment Method)** | The diagnosis of delirium is based on four clinical features: acute onset and fluctuating course, inattention, disorganized thinking, and altered level of consciousness. A score of 0 is assigned if the feature is not present, 1 if present at a mild level (for the level of consciousness item, this means the patient is either vigilant or lethargic), and 2 if present at a marked (moderate to severe) level (for the level of consciousness item, this means the patient is in stupor or coma). The total score ranges from 0 to 7, with 0 indicating normal, 1 mild, 2 moderate, and 3–7 severe delirium. |

**Supplementary Figure 1** Preferred Reporting Items for Systematic Reviews and Meta-Analysis (PRISMA) flowchart for search strategy and study selection.


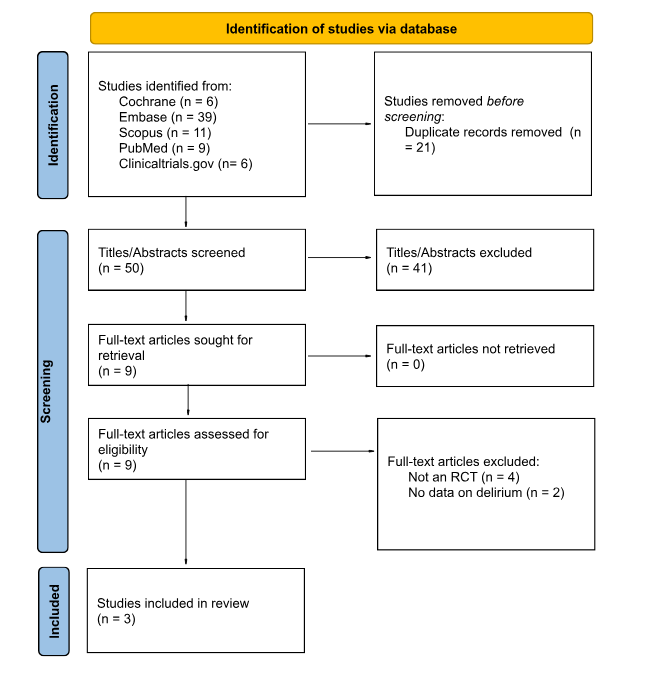


**Supplementary Figure 2** Forest plot of sensitivity analysis examining outcomes between patients in dexmedetomidine intervention and saline.


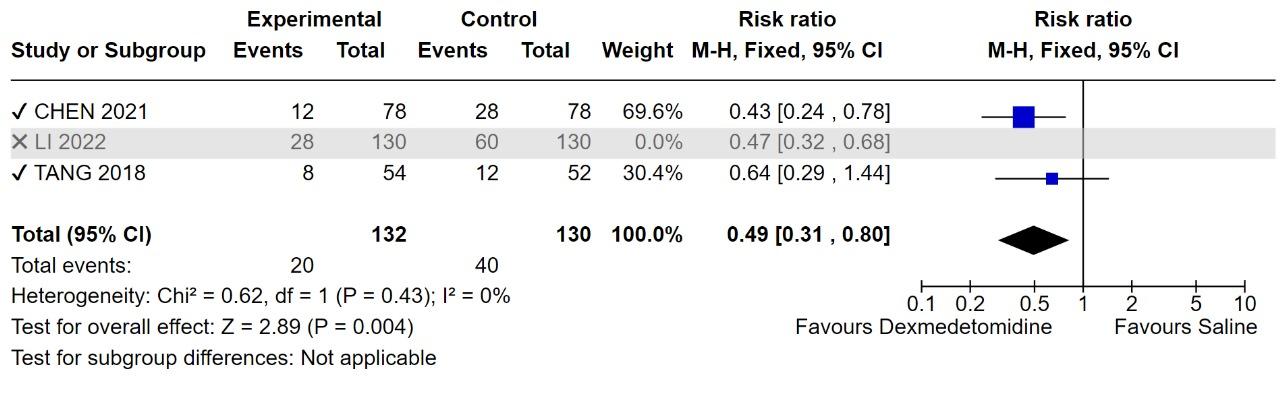

Supplement: Supplementary file 1 [file mmc1.docx]
